# Comparative study of rainfall prediction based on different decomposition methods of VMD

**DOI:** 10.1038/s41598-023-47416-x

**Published:** 2023-11-17

**Authors:** Xianqi Zhang, Qiuwen Yin, Fang Liu, Haiyang Li, Yu Qi

**Affiliations:** 1 Water Conservancy College, North China University of Water Resources and Electric Power, Zhengzhou, 450046 China; 2 Collaborative Innovation Center of Water Resources Efficient Utilization and Protection Engineering, Zhengzhou, 450046 China; 3 Technology Research Center of Water Conservancy and Marine Traffic Engineering, Zhengzhou, 450046 Henan Province China

**Keywords:** Climate sciences, Hydrology, Natural hazards

## Abstract

Rainfall forecasting is an important means for macro-control of water resources and prevention of future disasters. In order to achieve a more accurate prediction effect, this paper analyzes the applicability of the "full decomposition" and "stepwise decomposition" of the VMD (Variational mode decomposition) algorithm to the actual prediction service; The MAVOA (Modified African Vultures Optimization Algorithm) improved by Tent chaotic mapping is selected; and the DNC (Differentiable Neural Computer), which combines the advantages of recurrent neural networks and computational processing, is applied to the forecasting. The different VMD decompositions of the MAVOA-DNC combination together with other comparative models are applied to example predictions at four sites in the Huaihe River Basin. The results show that SMFSD (Single-model Fully stepwise decomposition) is the most effective, and the average Root Mean Square Error (RMSE) of the forecasts for the four sites of SMFSD-MAVOA-DNC is 9.02, the average Mean Absolute Error (MAE) of 7.13, and the average Nash-Sutcliffe Efficiency (NSE) of 0.94. Compared with the traditional VMD full decomposition, the RMSE is reduced by 7.42, the MAE is reduced by 4.83, and the NSE is increased by 0.05; the best forecasting results are obtained compared with other coupled models.

## Introduction

Generally speaking, the predictability of precipitation is derived from the "memory" of atmospheric circulation^1^. However, this "memory" typically lasts only a few weeks, which is insufficient for monthly precipitation predictions. With advancements in related fields, research has discovered the existence of "long memory" in oceanic and land surface processes that interact closely with atmospheric motion, offering the potential for monthly precipitation predictions^2^. Rainfall prediction is typically categorized into statistical methods relying on correlation relationships and physical-statistical methods based on physical factors. Nevertheless, these approaches face challenges in accurately capturing the inherent nonlinearity (complexity, diversity, and instability) of water anomalies and the uncertainties affecting precipitation anomalies due to the demanding data requirements and the uncertainty in detailed regional initial condition descriptions. In contrast, data-driven machine learning methods have shown promise in precipitation prediction, thanks to their powerful nonlinear mapping capabilities^3^. These methods bypass the intricate physical boundary conditions between influencing factors and precipitation, concentrating solely on identifying the optimal mathematical relationship between them. This renders machine learning-based monthly precipitation prediction methods highly practical and convenient. Among these methods, the decomposition-reconstruction-based machine learning model for monthly precipitation sequences offers distinctive convenience as it solely necessitates historical precipitation data as input to simulate future precipitation output. Narimani et al.^4^ used two methods, singular spectrum analysis (SSA) and empirical mode decomposition (EMD), to decompose the daily precipitation data, combined with light gradient-enhanced tree (LightGBM) and extreme gradient-enhanced tree (XGBoost) to make predictions. The VMD can adjust the number of modal decompositions on its own to effectively avoid modal aliasing^5^ and reduce the complexity of the sequence data, which is a commonly used and efficient time–frequency analysis tool with more advanced performance in the field of extracting effective information of precipitation sequences and unfolding sequence prediction research, and it has been verified to outperform the other decomposition methods in the relevant applications^6,7^. However, most of the previous machine learning prediction models based on VMD decomposition use full decomposition to construct the samples, which may result in mixing future precipitation information in the training samples, and thus the proposed method does not have the significance of real precipitation prediction. Therefore, some "stepwise decomposition" models for real forecasting are emerging, but there is little research on this method in the field of precipitation forecasting.

In addition, the prediction performance of the model is often affected by the hyperparameters set by human beings, and the intelligent optimization algorithm is one of the main means to prefer the hyperparameters. Adaptive Particle Swarm Algorithm (APSO) proposed by Zhang et al.^8^ optimizes the hyperparameters of Bidirectional Long Short-Term Memory Network (BiLSTM), obtaining higher prediction accuracy and faster convergence. Wang et al.^9^ proposed a combined wavelet decomposition-prediction-reconstruction model (WDPRM), which consists of a wavelet decomposition, Particle Swarm Optimized Support Vector Machines (PSO -SVM) and BP network optimized by Artificial Bee Colony Algorithm (ABC-BP) to obtain good prediction results. Mohammed et al.^10^ incorporated the Artificial Bee Colony algorithm, Particle Swarm Optimization, and Imperialist Competitive Algorithms to improve and optimize the internal parameters of the Artificial Neural Network (ANN) method for delivering a robust approach to identify non-linearities in rainfall patterns.

From the current research situation at both national and international level, the precipitation series are highly nonlinearized and noisy, and the use of data decomposition preprocessing techniques (EMD, SSA, Complementary Ensemble Empirical Mode Decomposition (CEEMD), and VMD, etc.) is a necessary means^11^. However, most of the researches directly use it for the decomposition and denoising of complete datasets (including training and test sets), and these decompositions and denoising algorithms often suffer from the "boundary effect", and the samples constructed in the training period may use the "future data information" which is unknown in reality, and the full decomposition may be difficult to satisfy the requirements of the actual forecasting; There are numerous different types of machine learning models, the traditional recurrent neural network structure is somewhat limited in the utilization of long-ago information (long memory), and the long short-term neural network (LSTM) requires a large amount of computational resources to process long-distance dependency sequences, in order to solve this problem, a variety of neural networks with an external storage mechanism have been designed, based on which Graves et al.^12^ have proposed the DNC, which has been improved on the Neural Turing Machine (NTM) based on the improvement of the storage management method, and the inclusion of temporal memory links allows the DNC to jump to read or update the memory information, thus combining the advantages of recurrent neural networks and computer processing. In addition, the hyperparameters of some machine learning models greatly affect the effect of precipitation prediction, and previous studies have mostly used particle swarm algorithms^13^ and genetic algorithms^14^ and other algorithms with limited ability to find the optimum, and it is necessary to further investigate the related new optimization algorithms.

Hence, this study scrutinizes the shortcomings of previous full decomposition-based precipitation prediction models for the Huaihe River Basin, which are not suitable for practical forecasting. Instead, it employs novel stepwise decomposition sample construction techniques that exclude future precipitation information in assessing real monthly precipitation prediction accuracy. Additionally, it harnesses the AVOA for refining the DNC hybrid learning neural network, incorporating initial population modification and parameter combination optimization search enhancements. The study concludes by comparing the forecasting accuracy of each coupled model and analyzing the optimal decomposition forecasting method, offering valuable insights for other precipitation forecasting research.

## Research methodology

### VMD with different decomposition methods

The rainfall time series data are relatively single, and the internal frequency information and change rule are difficult to be directly explored. Considering it as a nonlinear time series signal, the VMD algorithm is used to decompose it into a number of intrinsic mode functions (IMFs), so as to visualize the hidden information such as the inherent cyclical trend of the time series, and at the same time increase the amount of data information for the prediction model. However, in recent years, some studies in other fields have questioned the validity of this experimental framework, arguing that its true ability is "Hindcasting" rather than "Forecasting"^15,16^. The "full decomposition" approach uses the data information of the test period in the training period, which leads to the distortion of the model forecasting results. In this study, the applicability of the "full decomposition" and "stepwise decomposition" rainfall forecasting models in the Huaihe River Basin is compared.

#### Full decomposition of VMD

Variational mode decomposition (VMD) is a commonly used adaptive and fully recursive signal sequence processing method, which first requires the user to set two parameters—the number of decompositions K and the quadratic penalty factor α—and then iteratively search for the optimal solution corresponding to the model, which can adaptively match the optimal center frequency and finite bandwidth of each intrinsic mode function (IMF) and realize the effective separation of the IMFs, and the general IMF expression is:

1 $$ \begin{array}{*{20}c} {u_{k} \left( t \right) = A_{k} \left( t \right)cos\left[ {\varphi_{k} \left( t \right)} \right]} \\ \end{array} $$

Construction of the variational problem: Assuming that the original sequence f(t) is decomposed into K intrinsic IMFs, i.e., ${u}_{k}\left(t\right)$ with corresponding center frequencies and finite bandwidths and sparse, it is required that f (t) is equal to the sum of all ${u}_{k}\left(t\right)$ while pursuing the minimization of the sum of estimated bandwidths of each ${u}_{k}\left(t\right)$ i.e.:

2 $$ \begin{array}{*{20}c} {\mathop {\min }\limits_{{\left\{ {u_{k} } \right\}\left\{ {\omega_{k} } \right\}}} \left\{ {\mathop \sum \limits_{k} \partial_{t} \left\| {\left[ {\left( {\delta \left( t \right) + \frac{j}{\pi t}} \right)*u_{k} \left( t \right)} \right]e^{{ - j\omega_{k} t}} } \right\|_{2}^{2} } \right\}} \\ \end{array} { } $$

3 $$ \begin{array}{*{20}c} {s.t.\mathop \sum \limits_{k = 1}^{K} u_{k} = f\left( t \right)} \\ \end{array} $$

where: k is the number of modes to be decomposed (positive integer), $\left\{{u}_{k}\right\}$, $\left\{{\omega }_{k}\right\}$ correspond to the kth modal component and the center frequency after decomposition, respectively, $\delta \left(t\right)$is the Dirac function, $*$ is the convolution operator, and ${e}^{-j{\omega }_{k}t}$is the phase-volume representation of the center frequency in the complex plane.

(2) Solution of the variational model. To find the optimal solution for the above variational model, the quadratic penalty factor α and the Lagrange multiplier λ are used to construct the augmented Lagrange expression, which transforms the constrained variational model into an unconstrained variational model, denoted as follows:

4 $$ \begin{array}{*{20}c} {L\left( {\left\{ {u_{k} } \right\},\left\{ {\omega_{k} } \right\},\lambda } \right) = \alpha \mathop \sum \limits_{k = 1}^{K} \left\| {\partial_{t} \left[ {\left( {\delta \left( t \right) + \frac{j}{\pi t}} \right)*u_{k} \left( t \right)} \right]e^{{ - j\omega_{k} t}} } \right\|_{2}^{2} + \left\| {\mathop \sum \limits_{k = 1}^{K} u_{k} \left( t \right) - f\left( t \right)} \right\|_{2}^{2} + } \\ {\lambda \left( t \right),f\left( t \right) - \mathop \sum \limits_{k = 1}^{K} u_{k} \left( t \right)} \\ \end{array} $$

where $\alpha$ can be used to ensure that the reconstruction accuracy of the signal places a limit on the bandwidth. Using the alternating direction multiplier method, $\left\{{u}_{k}\right\}$, $\left\{{\omega }_{k}\right\}$ and $\lambda$ are updated alternately until the convergence condition is satisfied.

#### Stepwise decomposition of VMD

Constructing correct and efficient training test samples is the key point to serve the real precipitation prediction. In other medium- and long-term prediction fields, the more commonly used sample construction methods that can serve the real world are^17^: Semi-stepwise decomposition (SSD), Fully stepwise decomposition (FSD), Single-model semi-stepwise decomposition (SSD), Single-model FSD, and Single-model FSD), Fully stepwise decomposition (FSD), Single-mode SSD (SMSSD), and Single-mode SSD (SMSSD). Single-mode SSD (SMSSD) and Single-model FSD (SMFSD). The first two methods require K (K is the number of modal components) models to be constructed simultaneously. The latter two require only a single model to obtain the final prediction results, providing higher operational speed. The sample construction processes for FSD and SMFSD are described below, respectively.

The important thing about the FSD technique is that the initial sequence of samples to be decomposed is only an initial sequence of the length of the number of response variables, and then the test period samples are subsequently appended one by one to the training set for decomposition.

1. Sequence segments (S_1_, S_2_,…, S_m_) are decomposed to obtain K modal components, and the last m elements of each subsequence are extracted as explanatory variables.
2. The data S_m+1_ is appended to (S_1_, S_2_, …, S_m_), and the extended sequence segments (S_1_, S_2,_…, S_m_, S_m+1_) were decomposed, and the last m elements of each subsequence were extracted to continue to constitute the explanatory variables. By attaching new data one by one, the sequence segments are gradually expanded and the corresponding explanatory samples are extracted. For the last sample, the sequence segments (S_1_, S_2_, … S_N-1_), are decomposed and explanatory variables are extracted.
3. Response variables for all samples were extracted by decomposing the entire sequence segments (S_1_, S_2_, … S_N_), obtained by delaying the values of the corresponding one time period, and finally all the samples were partitioned into training and test sets.

**Figure 1:**
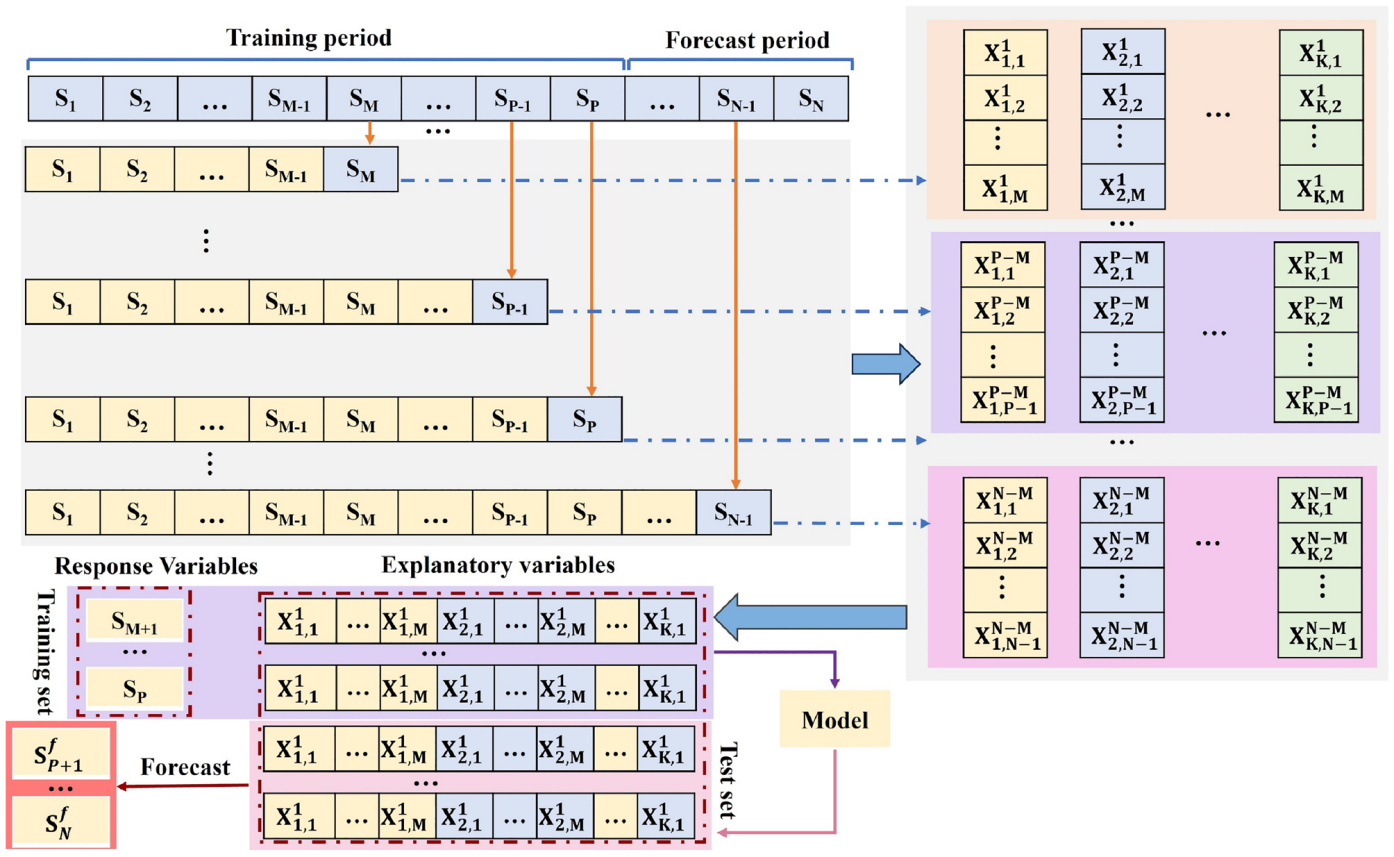
Sample construction process of SMFSD.

The biggest difference between SMFSD and FSD is that the precipitation results are directly used as the response variables instead of the results of each component, and the final summation and reconstruction are not needed, so the efficiency of the model operation is greatly improved. The construction process of SMFSD is shown in Fig. 1.

1. The original precipitation series (S_1_, S_2_, … S_N_) is divided into the training period (S_1_, S_2_, …, S_p_) and test period (S_p1_, S_p+2_, …, S_N_).
2. Sequence segments (S_1_, S_2_, …, S_m_) were decomposed into K subsequences, and the last m elements of each subsequence were extracted as explanatory variables for the first sample, while the response variable was Sm + 1. Then, the sequence segments (S_1_, S_2_, …, S_m_, S_m+1_) is decomposed into K subsequences, and so on, until the sequence segment (S_1_, S_2_, …, S_m_, S_m+1_, S_N-1_) are decomposed. Finally, N-m samples can be obtained. The first P-m samples are used as the training set and the remaining samples are used as the test set. Since the response variable is the actual precipitation data, SMFSD also requires only one model to be trained.

### Modified AVOA

#### AVOA

African Vultures Optimization Algorithm (AVOA) is an optimization algorithm proposed by Abdollahzadeh et al.^18^ in, which has shown better optimization results in relevant tests compared to the Teaching and Learning Optimization Algorithm (TLBO), Gray Wolf Optimization Algorithm (GWO), Particle Swarm Algorithm (PSO) and Differential Evolutionary Algorithm (DE). The AVOA algorithm defines the objective as the vulture's hunger level: the worse the fitness value is, the vulture feels hungry and tries to search for food, which is the reality of the algorithm's optimization process. The AVOA iteratively proceeds as follows.

1. Phase 1. After randomly initializing the population position, the optimal or suboptimal individuals are selected according to the "roulette" rule for the next stage of the search. For the ith vulture in the population, the selection of its learning object can be expressed as follows:
  5 $$ \begin{array}{*{20}c} {R_{\left( i \right)} = \left\{ {\begin{array}{*{20}l} {{\text{Optimal}} \,{\text{Vulture}},\,{\text{if}}\,{\text{rand}} \le {\text{L}}} \hfill \\ {{\text{Suboptimal }}\,{\text{Vulture}},\,{\text{else}}} \hfill \\ \end{array} } \right.} \\ \end{array} $$
  where, L is a user-defined parameter, located between (0,1), when L tends to 0, it contributes to the increase of population diversity, and vice versa, it accelerates the aggregation of the population; rand is a [0,1] uniformly distributed random number.
2. Phase 2: Define the starvation rate to realize the conversion of algorithm development and exploration process. Vultures start to search for food because of hunger, but if they are hungry, they don't have enough energy to fly for a long time over a long distance, and they can only search for food next to strong vultures to save energy. Therefore, the AVOA algorithm defines a starvation rate F as shown in Eq. (7), which increases as the iterative process progresses in order to facilitate the development process more likely.
  6 $$ \begin{array}{*{20}c} {t = h \times \left[ {sin^{w} \left( {\frac{\pi }{2} \times \frac{{i_{iter} }}{{maxi_{iters} }}} \right) + \cos \left( {\frac{\pi }{2} \times \frac{{i_{iter} }}{{maxi_{iters} }}} \right) - 1} \right]} \\ \end{array} $$
  7 $$ \begin{array}{*{20}c} {F = \left( {2 \times rand + 1} \right) \times z \times \left( {1 - \frac{{i_{iter} }}{{maxi_{iters} }}} \right) + t} \\ \end{array} $$
  where, h, z are uniformly distributed random numbers in the interval [−2,2], [−1,1] respectively; w is a user-defined parameter that controls the probability that the algorithm enters the exploration mode in the final stage; i_iter_ is the current number of iterations in the course of the algorithm.
3. Phase 3: Spatial exploration. In this process, the AVOA algorithm uses a user-defined parameter P to determine what kind of exploration mode to enter, taking a value between (0,1). This phase is executed according to Eq. (8) to Eq. (9):
  8 $$ \begin{array}{*{20}c} {P\left( {i + 1} \right) = \left\{ {\begin{array}{*{20}l} {R\left( i \right) - D\left( i \right)F,if P_{1} \ge rand} \hfill \\ {R\left( i \right) - F + rand \times \left[ {\left( {ub - lb} \right) \times rand_{3} + lb} \right],if P_{1} < rand} \hfill \\ \end{array} } \right.} \\ \end{array} $$
  9 $$ \begin{array}{*{20}c} {D\left( i \right) = \left| {X \times R\left( i \right) - P\left( i \right)} \right|} \\ \end{array} $$
  where, P(i + 1) is the updated position of the vulture; X is [0,2] uniformly distributed random number.
4. Phase 4: Localized exploitation. When the absolute value of the starvation rate $\left|F\right|<1$, the AVOA algorithm initiates the exploitation phase. Unlike the exploration phase, this phase contains two types of subphases, and the initiation demarcation of the two subphases is $\left|F\right|= 0.5.$

Subphase 1 judgment condition: $\left|F\right|\ge 0.5$. In this phase, the position updating method mimics the characteristics of vulture spiral flight, and is executed according to Eqs. (10–13):

10 $$ \begin{array}{*{20}c} {P\left( {i + 1} \right) = \left\{ {\begin{array}{*{20}l} {D\left( i \right) \times \left( {F + rand} \right) - d\left( t \right),if P_{2} \ge rand} \hfill \\ {R\left( i \right) - \left( {S_{1} + S_{2} } \right),if P_{2} < rand} \hfill \\ \end{array} } \right.} \\ \end{array} $$

11 $$ \begin{array}{*{20}c} {d\left( t \right) = R\left( i \right) - P\left( i \right)} \\ \end{array} $$

12 $$ \begin{array}{*{20}c} {S_{1} = R\left( i \right) \times \left( {\frac{{rand_{5} \times P\left( i \right)}}{2\pi }} \right) \times \cos \left[ {P\left( i \right)} \right]} \\ \end{array} $$

13 $$ \begin{array}{*{20}c} {S_{2} = R\left( i \right) \times \left( {\frac{{rand_{6} \times P\left( i \right)}}{2\pi }} \right) \times \sin \left[ {P\left( i \right)} \right]} \\ \end{array} $$

where the parameter P_2_ takes values between (0,1).

Substage 2 judgment condition: $\left| F \right| < 0.5$ This stage is executed according to Eqs. (14–16):

14 $$ \begin{array}{*{20}c} {P\left( {i + 1} \right) = \left\{ {\begin{array}{*{20}l} {\frac{{A_{1} + A_{2} }}{2},if P_{3} \ge rand_{{P_{3} }} } \hfill \\ {R\left( i \right) - \left| {d\left( t \right)} \right| \times F \times Levry\left( d \right),if P_{3} < rand_{{P_{3} }} } \hfill \\ \end{array} } \right.} \\ \end{array} $$

15 $$ \begin{array}{*{20}c} {A_{1} = {\text{Optimal Vulture}}\left( i \right) - \frac{{{\text{Optimal Vulture}}\left( i \right) \times P\left( i \right)}}{{{\text{Optimal Vulture}}\left( i \right) - P\left( i \right)^{2} }} \times F} \\ \end{array} $$

16 $$ \begin{array}{*{20}c} {A_{2} = {\text{Suboptimal Vulture}}\left( i \right) - \frac{{{\text{Suboptimal Vulture}}\left( i \right) \times P\left( i \right)}}{{{\text{Suboptimal Vulture}}\left( i \right) - P\left( i \right)^{2} }} \times F} \\ \end{array} $$

Similarly, the parameter P3 takes values between (0,1).

#### Tent chaotic mapping initialization of populations

Excellent population initialization can speed up the convergence of the population and improve the performance of the system. The chaotic sequence generated by chaotic mapping has the characteristics of good traversal and strong randomness^19^, which can be used to replace [0,1] uniformly distributed random numbers to generate diverse populations. Therefore, choosing suitable chaotic sequences for population initialization is a key issue at this stage, and the use of chaotic sequences to initialize algorithmic populations is widely used at present^20,21^.Tent chaotic mapping^22^ with randomness, ergodicity, and regularity characteristics of chaotic sequences in large quantities, is one of the means commonly used to improve the representativeness of the initial population of optimization algorithms, and has been verified by examples to have better traversal than classical chaotic mapping^23,24^. The mathematical model of standard Tent mapping is as follows:

17 $$ \begin{array}{*{20}c} {x_{n + 1} \left\{ {\begin{array}{*{20}l} {2x_{n} + rand \times \frac{1}{N},0 \le x_{n} \le \frac{1}{2}} \hfill \\ {2\pi \left( {1 - x_{n} } \right) + rand \times \frac{1}{N},\frac{1}{2} \le x_{n} \le 1} \hfill \\ \end{array} } \right.} \\ \end{array} $$

Zhang et al.^25^ considered that there might be small and unstable periodic points in the classical Tent series and introduced a random variable considering the population size N into Eq. (17) to obtain the improved Tent mapping.

In using the above improved Tent mapping, it is found that the obtained chaotic variables are easy to go beyond the [0,1] boundary when the population size is small. Therefore, $N=100$ is taken uniformly in this paper.

Chaotic mapping initialization population method

According to the characteristics of Tent chaotic mapping, the steps of generating N chaotic individuals within the feasible domain are as follows.

- Step 1: According to the number of dimensions D, take random initial values X_1i_, X_2i_, …, X_Di_, $i=1$ in the interval [0,1].
- Step 2: Iterative calculation according to Eqs. (5) and (7), $i= i+1$, will produce D sequences of X_j_ $j=1:D$.
- Step 3: Stop iteration when i equals to the maximum number of iterations, each X sequence corresponds to each dimension to produce N chaotic individuals.

#### Selection of suboptimal vultures

In the standard AVOA algorithm, the first and second ranked vultures in the population in terms of fitness values will serve as the optimal and suboptimal vultures as potential learning objects for the population during the next iteration. However, the optimal and suboptimal vultures tend to be closer together in the later stages of the population iteration, resulting in the algorithm having been bound by a local optimum. Therefore, considering strengthening the ability of the AVOA to escape from local optimality at the later stage, this paper proposes that the vulture with the third highest fitness ranking or even any vulture in the population can potentially be used as a learning object. On this basis there is the following mathematical model.$rr=0.9+0.1*chaos\left(iter\right);$

18 $$ \begin{array}{*{20}c} {if\,rand < rr} \\ \end{array} $$

Suboptimal vultures = second best individual for fitness value.

                                             else

Strategy 1: Suboptimal vultures = any but the best and next best individual.

                                                     end

In the above suboptimal vulture selection model, chaos represents the chaotic sequence generated by the Tent mapping with the length of Maximum number of iterations; $$iter$$ is the current number of iterations; and rand represents the random number generated by [0,1] uniform distribution. Whenever a suboptimal vulture is to be selected, a chaotic number $$rr $$greater than 0.9 is first generated and then selected according to the model. Therefore, there is a lower probability of selecting vultures with weaker fitness values, and only vultures with the second highest fitness value are considered as suboptimal vultures for the initialization of the population using the Tent chaotic mapping. Thus, the exploration capability of the algorithm is improved without significant loss of exploitation capability.

In addition, the algorithm's own parameters also have a profound effect on the algorithm's performance. The standard African vulture optimization algorithm is parameterized with a step size of 0.1 for its four parameters L, P1, P2, and P3, resulting in a recommended parameter combination. In this study, a finer step size (0.05) was set for this set of parameter combinations, details of which can be found in Table 1.

**Table 1:** Parameter design of the optimization algorithm.

Algorithm	Initialization method	Suboptimal vulture selection strategy	L	P_1_	P_2_	P_3_
AVOA	Uniform distribution on [0,1]	The second-best individual in terms of fitness	0.8	0.6	0.4	0.6
MAVOA	Tent chaotic map	Formula (18) Strategy 1	0.8	0.6	0.4	0.55

### DNC

DNC hybrid learning neural network, is a special kind of recurrent neural network with external memory (not trainable). At each time step t, a trainable controller decides the final output prediction by linearly combining the prediction information of the two parts after exchanging the information flow with the external memory based on the information flow at the moment t − 1.

**Figure 2:**
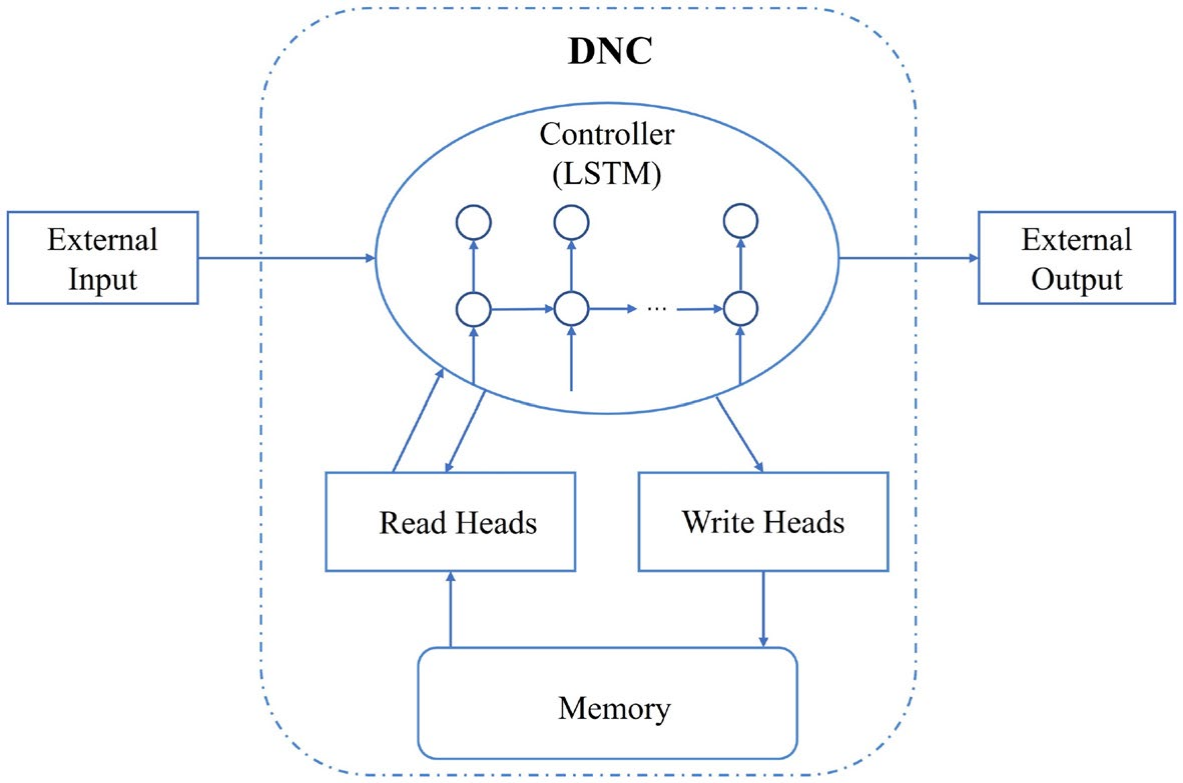
DNC model structure.

Figure 2 illustrates the overall architecture of the DNC model. Input data is processed within the controller, and the generated memory information is written to the memory bank. The interaction between the controller and the memory bank is facilitated by the read and write heads.

1. Controller $${\varvec{\eta}}$$

The controller $\eta$ in the DNC is a variant of the LSTM. at some time, the controller $\eta$ obtains an input vector ${x}_{t}$, Read R vectors $r_{t - 1}^{1} ,r_{t - 1}^{2} , \ldots r_{t - 1}^{R}$ in the memory storage matrix ${M}_{t-1}$ Thus, the vector ${x}_{t}$ is concatenated with the R readout vectors, and the input to the controller $\eta$ at time t is $x_{t} = \left[ {x_{t} ;r_{t - 1}^{1} ,r_{t - 1}^{2} , \ldots r_{t - 1}^{R} } \right]$. At moment t, the output value of the hidden layer for an LSTM network with 1 hidden layer is:

19 $$ \begin{array}{*{20}c} {h^{l}_{t} = LSTM\left( {x_{t} } \right)} \\ \end{array} $$

At each moment t, the controller $\eta$ computes the output vector $v_{t}$ and an interaction vector $\xi_{t}$, defined as follows:

20 $$ \left\{ {\begin{array}{*{20}l} {v_{t} = W_{y} [h_{t}^{1} ;h_{t}^{2} ; \ldots ;h_{t}^{L} ]} \hfill \\ {\xi_{t} = W_{\xi } [h_{t}^{1} ;h_{t}^{2} ; \ldots ;h_{t}^{L} ]} \hfill \\ \end{array} } \right. $$

The controller $\eta$ passes the information back by creating a loop in the computational graph, which in turn yields ${v}_{t}$ etc.^26^

Finally, the $\eta$ output vector is as follows.

21 $$ \begin{array}{*{20}c} {y_{t} = v_{t} + W_{r} [r_{t}^{1} ;r_{t}^{2} ; \ldots ;r_{t}^{R} ]} \\ \end{array} $$

(2) Read and write head and memory

The controller η operates on the data in the memory through the read/write head. The read or write positions are determined by the corresponding weights, and the set of allowed weights at N positions is the non-negative quadrant of the standard simplex form in R^N^.

22 $$ \begin{array}{*{20}c} {\Delta_{N} = \left\{ {{\varvec{\alpha}} \in {\varvec{R}}^{{\varvec{N}}} :\alpha_{i} \in [0,1],\,\mathop \sum \limits_{i = 1}^{N} \alpha_{i} \le 1} \right\}} \\ \end{array} $$

In the read operation, multiple read weights $w_{t}^{r,1} ,w_{t}^{r,2} , \ldots ,w_{t}^{r,R} \in {\Delta }_{N}$ are used to compute the weighted average of the contents, thus defining the read vector as follows.

23 $$ \begin{array}{*{20}c} {r_{t}^{i} = M_{t}^{{\text{T}}} w_{t}^{r,i} } \\ \end{array} $$

In the write operation, the write weight ${\text{w}}_{{\text{t}}}^{W} \in {\Delta }_{{\text{N}}}$ is combined with the erase vector $e_{t}$ and the write vector $v_{t}$ to modify the memory storage matrix.

24 $$ \begin{array}{*{20}c} {M_{t} = M_{t - 1}^{ \circ } \left( {E - w_{t}^{{\text{w}}} e_{t}^{{\text{T}}} } \right) + w_{t}^{{\text{w}}} v_{t}^{{\text{T}}} } \\ \end{array} $$

Here: ° denotes the Hadamard product; E is the All 1 matrix of N × M.

(3) Time-series memory link

The temporal memory link holds information about the order in which the memory locations are written and is denoted as ${L}_{t}$. ${L}_{t}\left[i,j\right]$ indicates the degree to which location i is written after location j is written, and each row and column of ${L}_{t}$ can be defined with location weights.

25 $$ \begin{array}{*{20}c} {L_{t} \left[ {i,j} \right] = \left( {1 - w_{t}^{{\text{w}}} \left[ i \right] - w_{t}^{{\text{w}}} \left[ j \right]} \right)L_{t - 1} \left[ {i,j} \right] + w_{t}^{{\text{w}}} \left[ i \right]p_{t - 1} \left[ j \right]} \\ \end{array} $$

Here, ${p}_{t}$ is the priority weight and ${p}_{t}\left[i\right]$ denotes the degree to which position i was written last.

26 $$ \begin{array}{*{20}c} {p_{t} = \left( {1 - \mathop \sum \limits_{i} w_{t}^{{\text{w}}} \left[ i \right]} \right)p_{t - 1} + w_{t}^{{\text{w}}} } \\ \end{array} $$

For read head $i$, define reverse weights ${f}_{t}^{i}$ and forward weights ${b}_{t}^{i}$:

27 $$ \begin{array}{*{20}c} {f_{t}^{i} = L_{t} w_{t}^{{{\text{r}},{\text{i}}}} } \\ \end{array} $$

28 $$ \begin{array}{*{20}c} {b_{t}^{i} = L_{t}^{T} w_{t}^{{{\text{r}},{\text{i}}}} } \\ \end{array} $$

### Model coupling

**Figure 3:**
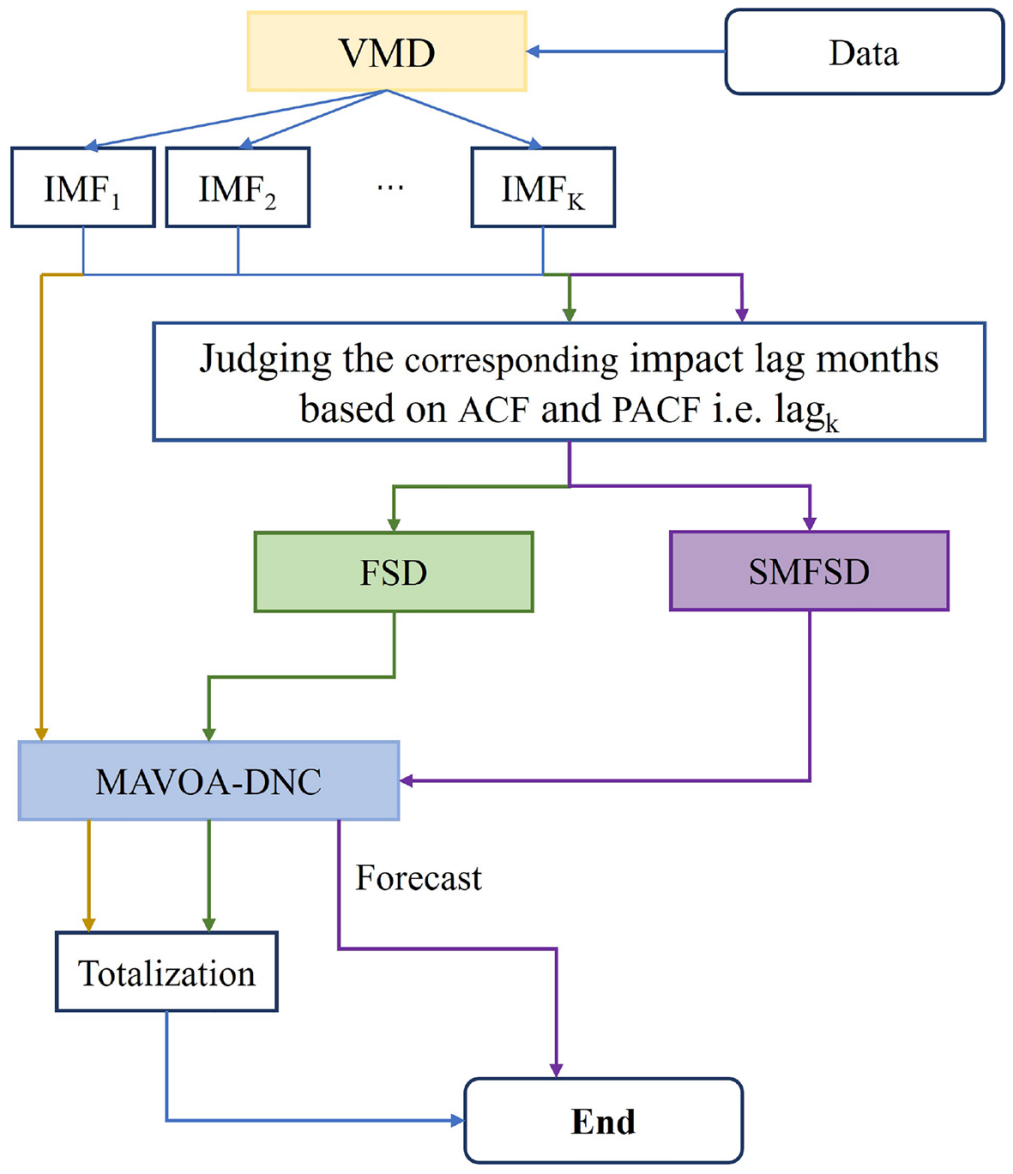
The combined SMFSD-MAVOA-DNC model prediction flow chart.

- Step 1: K modal components are obtained by decomposing the precipitation sequence using the VMD algorithm according to different stepwise decomposition sample construction methods.
- Step 2: In order to more accurately describe the number of pre-influence factors of each component, for the kth modal component according to the ACF and PACF to determine the corresponding influence lag month lag_k_ taking Huaian as an example, 6 modal components are obtained, and the lag month of each component is lag_k_ = [2,7,4,6,6,3].
- Step 3: Training and testing samples were generated using various stepwise decomposition methods, with a training-to-prediction sample ratio of 19:2, and normalization was applied based on the training samples.
- Step 4: The training samples are input into the MAVOA-DNC model for validation according to the requirements of different stepwise decomposition sample construction methods. The labels of the test samples are fed into the trained specific model to get the test sample predictions.
- Step 5: If the samples are constructed for the VMD and FSD methods, the cumulative value after the prediction of each component needs to be calculated, which is the final prediction result. The coupled model for precipitation prediction is shown in Fig. 3.

## Case study analysis

### Data source

**Figure 4:**
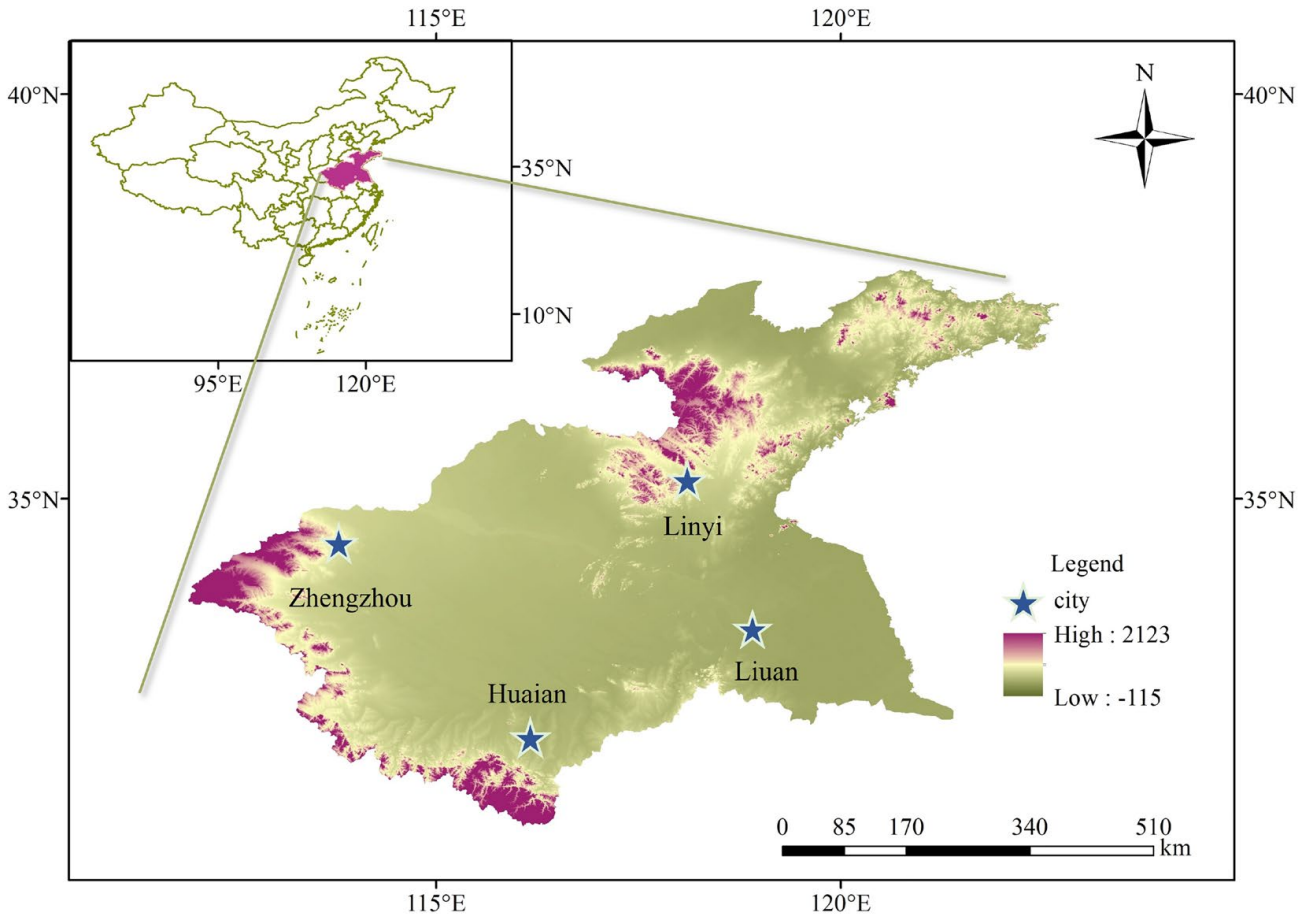
Location map of the study area.

The Huaihe River Basin is situated between the basins of the Yangtze River and Yellow River and is located at 111° 55′–121° 25′ east longitude and 30° 55′–36° 36′ north latitude, covering an area of 270,000 km^2^. The Huaihe River basin is located in the climate transition zone between the north and south of China, and belongs to the warm temperate semi-humid monsoon climate zone, with the extreme maximum temperature reaching 44.5℃ and the extreme minimum temperature reaching  − 24.1℃. The distribution of precipitation in the Huaihe River basin is extremely uneven within the year, with a lot of rain in summer and little rain in winter, the average annual precipitation in the Huaihe River basin is approximately 920 mm, with an average surface water evaporation ranging from 900 to 1500 mm. It is characterized by drought with limited rainfall in winter and spring, sultry conditions with heavy rainfall in summer and autumn, and significant fluctuations in temperature and precipitation, resulting in frequent droughts and floods. The specific location of the study area is shown in Fig. 4, which was created using ArcMap 10.7, accessible at the following URL: www.arcgis.com.

**Figure 5:**
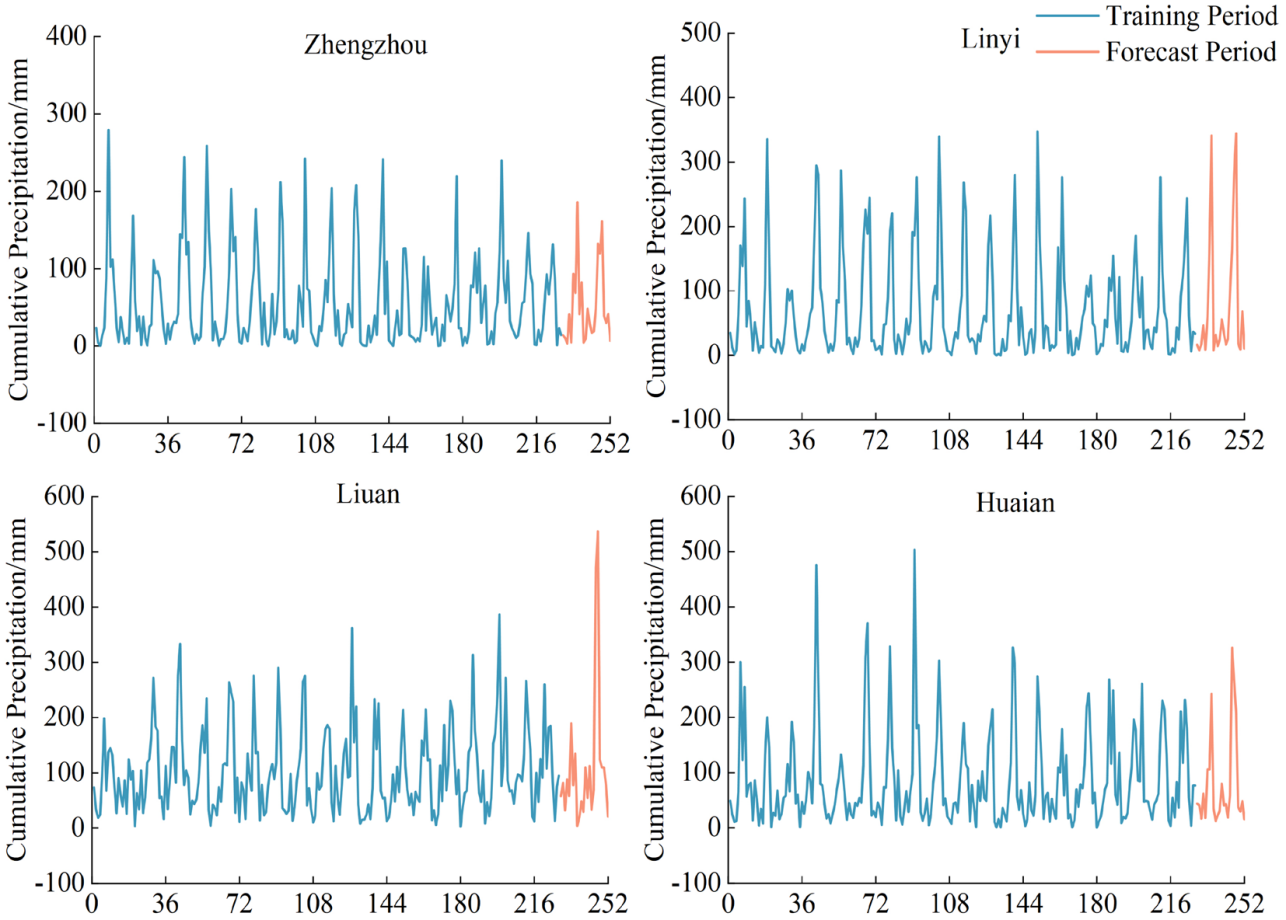
Monthly precipitation data of Zhengzhou Linyi Liuan and Huaian from 2000 to2020.

The meteorological data in this paper were adopted from the National Meteorological Information Center of the China Meteorological Administration (http://data.cma.cn) on the monthly precipitation of meteorological stations in Zhengzhou, Linyi, Liuan and Huaian cities dataset (2000–2020). The study data are shown in Fig. 5.

From Fig. 5 and Table 2, it can be observed that the distribution of rainfall in the study area is extremely uneven throughout the year, and there are abrupt changes in the data over time. This presents a significant challenge in constructing prediction models. Therefore, it is essential to utilize VMD with different decomposition methods to uncover the internal patterns of rainfall time series and couple them with advanced rainfall prediction models to enhance prediction accuracy.

**Table 2:** Monthly rainfall data summary table for various cities.

City	Mean	Standard deviation	Maximum value	Minimum value
Zhengzhou	53.93	58.24	279.29	0.00
Linyi	69.05	80.57	347.51	0.01
Liuan	102.17	91.92	536.81	3.08
Huaian	84.76	85.49	503.06	0.73

### Decomposition of data

**Figure 6:**
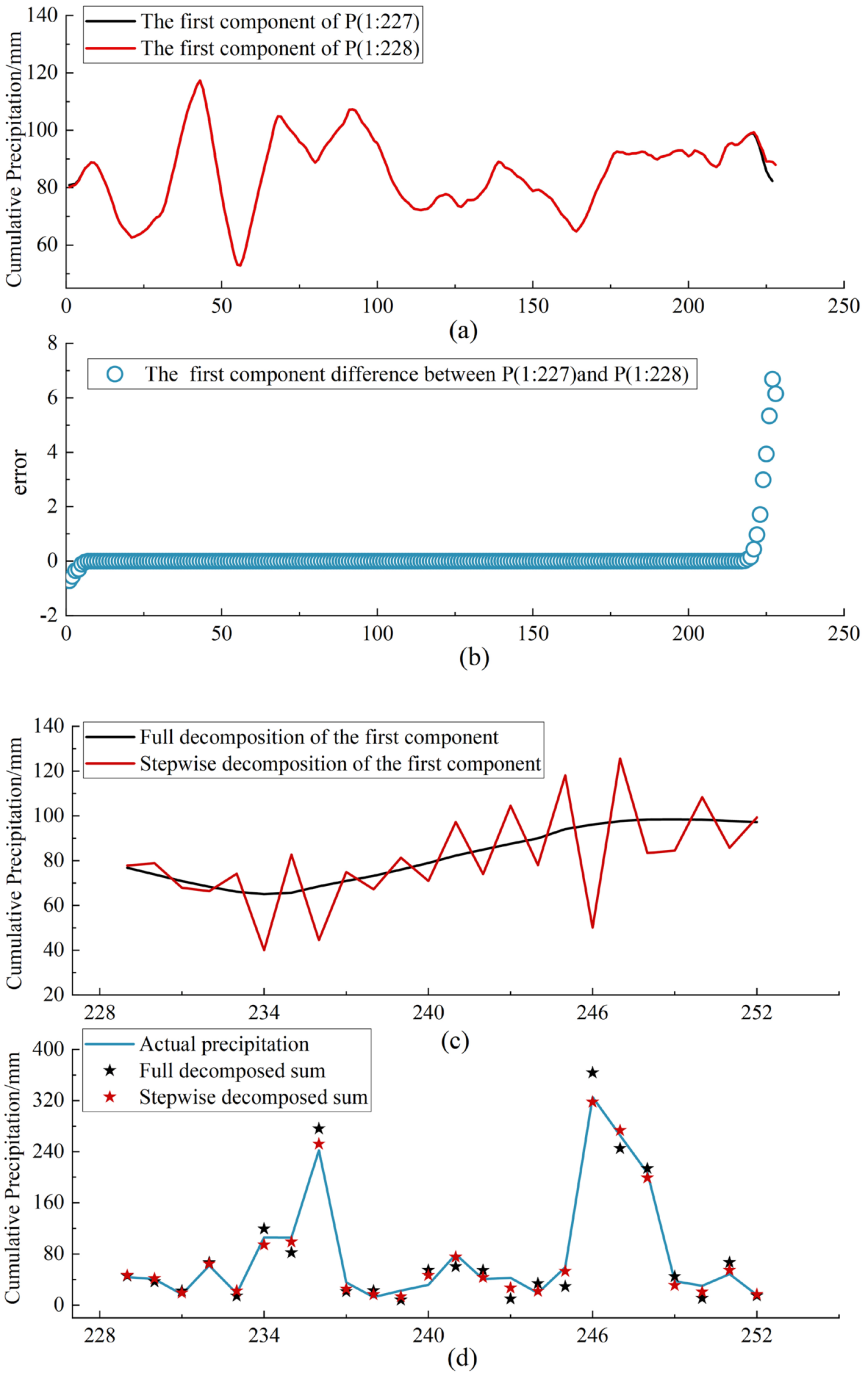
Boundary effects of stepwise decomposition.

The decomposition of the whole precipitation time series at one time will bring the information that can only be known in the future into the model training process, which will lead to the distortion of the prediction. However, due to the "boundary effect", decomposing the training set and test set separately often leads to poor prediction results, so the "stepwise decomposition" method is more scientific and reasonable. In order to better compare the prediction effects of "full decomposition" and "stepwise decomposition", the monthly precipitation series of the city of Huaian is used to analyze the decomposition effects of the two methods, and the analysis results are shown in Fig. 6.

In the actual precipitation prediction work, the past historical data belong to the training period, and the future results are the testing period. The first 227 samples of Huai'an precipitation series and the first 228 samples of the same station are selected, i.e., P (1:228) is obtained by adding one month of precipitation data to P (1:227) to simulate the actual precipitation prediction.

Using the VMD decomposition of P (1:228) and P (1:227) with the same parameters, subfigure (a) in Fig. 6 shows the first component in the respective decomposition results. P (1:228) and P (1:227) are identical except for the last sample, so the red and black lines in subfigure (a) should coincide, but they are separated at the all-important right-hand boundary. Fundamentally, the red line represents the actual first component, i.e., the result of the full decomposition, while the black line represents the first component obtained in the actual application, which is clearly not equal. Therefore, from the point of view of additional sensitivity, the full decomposition method cannot reflect the actual application results, and subfigure (b) also reflects this difference. Extending this additional sensitivity to a longer test period, subfigure (c) further illustrates the decomposition difference between the full decomposition and the additional step-by-step decomposition, which can be seen to follow the entire test period. In summary, the "full decomposition" approach cannot serve the actual precipitation prediction. In addition, when the modal components are superimposed to obtain the reconstruction difference between the full decomposition and the stepwise decomposition in subfigure (d), it can be seen that the reconstruction result of the stepwise decomposition represented by the red line has a smaller difference with the actual precipitation and has a lower impact on the subsequent prediction.

### Rainfall forecast

**Figure 7:**
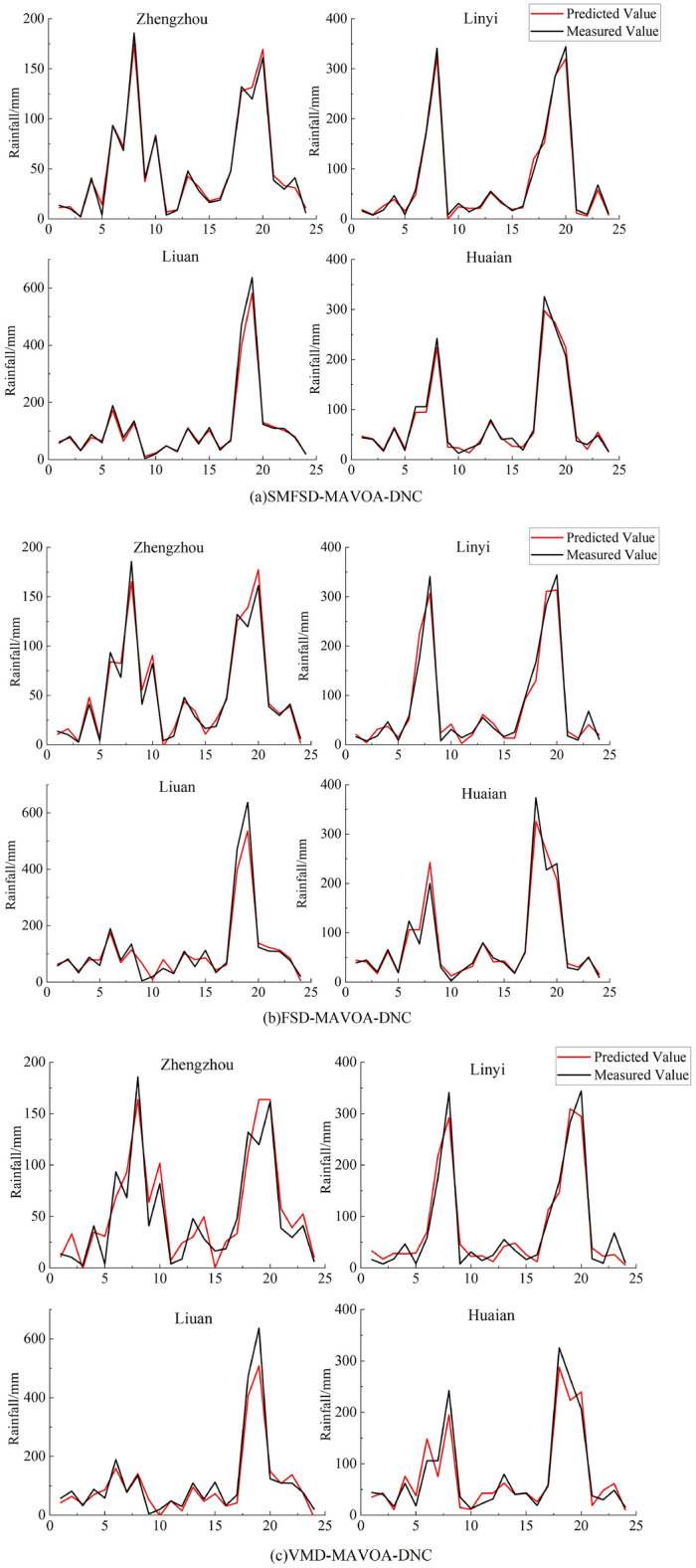
Model prediction curve.

In order to further analyze the suitability of different decomposition methods for coupling with the prediction models, three sets of coupled models consisting of the "full decomposition", "FSD" and "SMFSD" methods of VMD are used to predict the monthly rainfall data of Zhengzhou city, Linyi city, Luan city and Huai'an city in 20 years. Next, the "full decomposition", "FSD" and "SMFSD" methods of VMD are used to predict the monthly rainfall data of Zhengzhou City, Linyi City, Liuan City and Huaian City in 20 years, so as to identify more scientific and reasonable coupled models with higher prediction accuracies. The rainfall from 2000 to 2018 was used as the training period, and the rainfall from 2019 to 2020 was used as the test period. The prediction results of different coupled models are shown in Fig. 7.

The figure shows the prediction results of the coupled models with different decompositions for the four cities, and it can be seen that the prediction accuracy of the three forecasting models is lower in the flood season of June–September, when the rainfall varies greatly. Among them, the rainfall in June–September 2020 in Liuan City changes drastically compared with the previous years, so the forecast results of the three coupled models here all have large errors. In contrast, the coupled model with SMFSD decomposition in subfigure (a) predicts the rainfall most closely with the actual rainfall; the coupled model with FSD decomposition in subfigure (b) can predict the overall trend of rainfall changes, but the forecasting accuracy is lower than that in subfigure (a) at the peak of rainfall, and the coupled model with full decomposition in subfigure (c) does not adequately explore and has a certain error in the cyclical trend of the rainfall time series during decomposition. The coupled model with full decomposition in subplot (c), due to the insufficient excavation of the cyclic trend of rainfall time series during the decomposition and the existence of certain errors, has a greater impact on the subsequent forecast, and can only roughly forecast the trend of rainfall, and the overall forecasting accuracy is obviously lower than that of the previous two.

**Figure 8:**
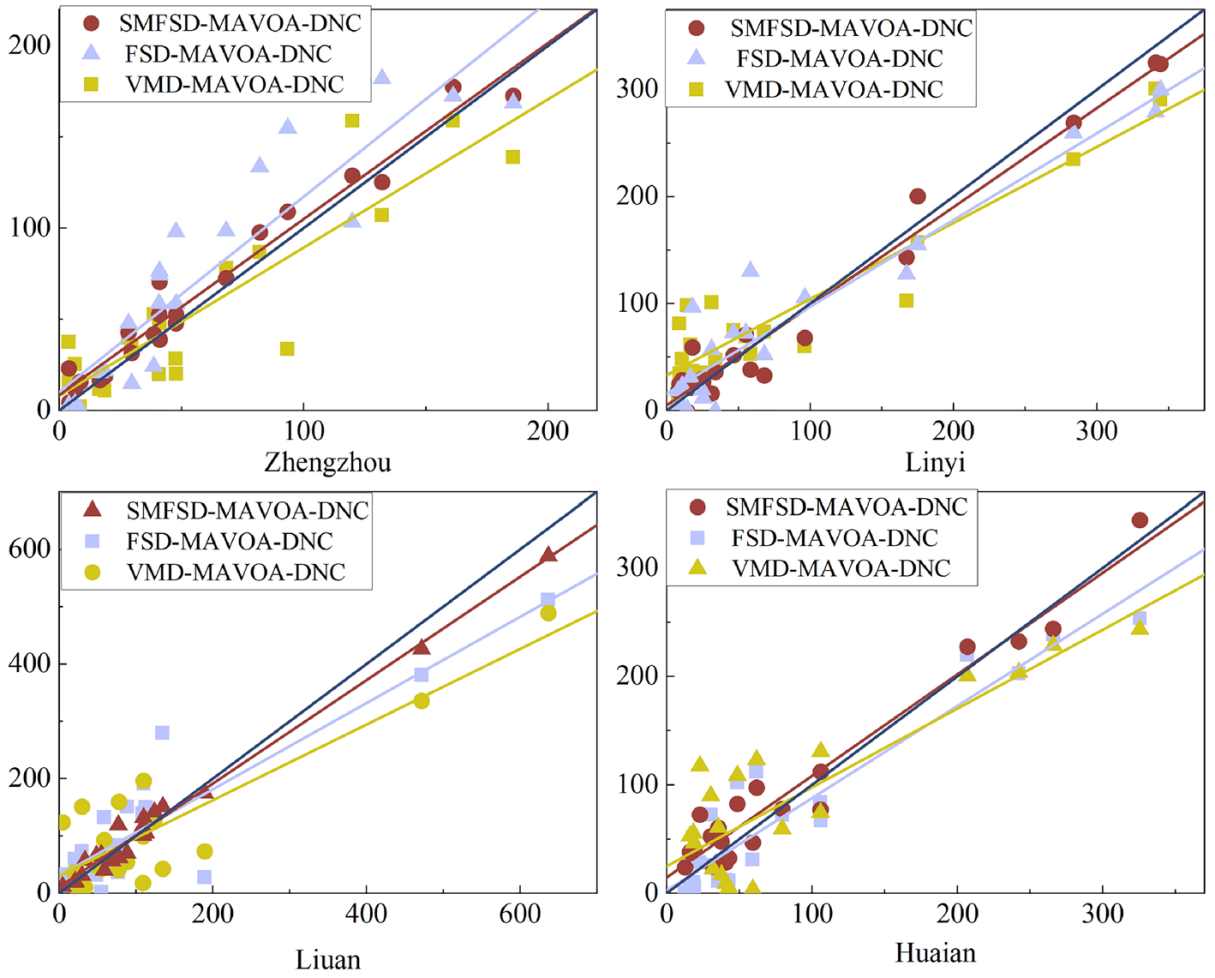
Comparison of the linear fitting effects of the models.

In order to more intuitively show the prediction effect of the three different decomposition modes of the coupled model on each city, the linear fitting effect of the three is compared as in Fig. 8, and the average prediction accuracies of each model for the four city predictions are shown in Table 3.

29 $$ \begin{array}{*{20}c} {RMSE = \sqrt {\frac{1}{n}\mathop \sum \limits_{i = 1}^{n} \left( {\sigma_{i} - \sigma_{0} } \right)^{2} } } \\ \end{array} $$

30 $$ \begin{array}{*{20}c} {MAE = \mathop \sum \limits_{i = 1}^{n} \left| {\frac{{\left( {\sigma_{i} - \sigma_{0} } \right)}}{n}} \right|} \\ \end{array} $$

31 $$ \begin{array}{*{20}c} {NSE = 1 - \frac{{\mathop \sum \nolimits_{i = 1}^{n} \left( {\sigma_{i} - \sigma_{0} } \right)^{2} }}{{\mathop \sum \nolimits_{i = 1}^{n} \left( {\sigma_{0} - \sigma } \right)^{2} }}} \\ \end{array} $$

**Table 3:** Average prediction accuracy of each model.

City	Model	RMSE	MAE	NSE
Average value	VMD-MAVOA-DNC	16.44	11.96	0.86
	FSD-MAVOA-DNC	13.92	10.76	0.89
	SMFSD-MAVOA-DNC	9.02	7.13	0.94

Here, ${\sigma }_{i}$ is the predicted value at time $i$; ${\sigma }_{0}$ is the measured value at time $i$; $\sigma$ is the mean value of the measured value^27–30^.

**Figure 9:**
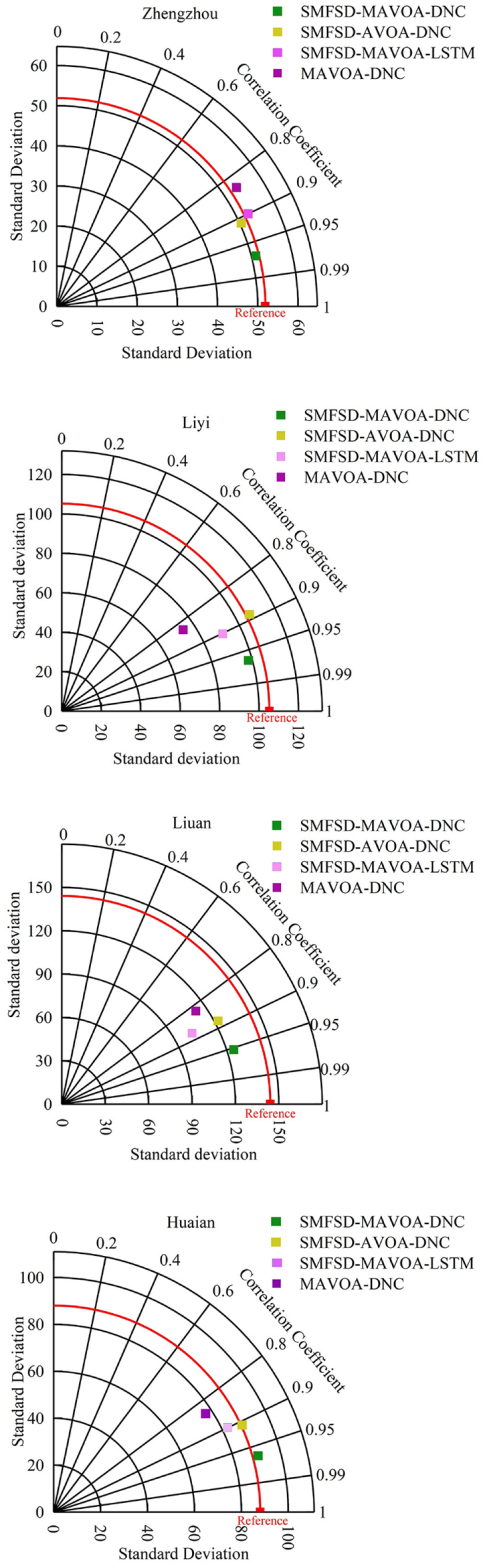
Model relative error comparison chart.

As can be seen from Fig. 9, the forecasting accuracy of the three coupled models is generally low for the city of Luan due to the presence of the sudden 2020 rainstorm in the city; compared to the other three cities, the forecasting effect is best for the city of Zhengzhou, which has a smoother trend in the rainfall time series. For all cities, the forecasting effect is SMFSD-MAVOA-DNC > FSD-MAVOA-DNC > VMD-MAVOA-DNC, Similarly, the results in Table 3 show that SMFSD-MAVOA-DNC has the highest average forecasting accuracy, and compared with the traditional VMD decomposition, the RMSE decreases by 7.42, the MAE decreases by 4.83, and the NSE increases by 0.05. NSE increased by 0.05, which fully indicates that the decomposition method of SMFSD is more suitable for actual rainfall forecasting.

## Discussion

In order to further verify the prediction effect of the coupled models, four models, SMFSD-MAVOA-DNC, SMFSD-AVOA-DNC, SMFSD-MAVOA-LSTM, and MAVOA-DNC, are selected to conduct experiments in turn. Figure 9 shows the comparison of the prediction errors of each model for the four cities.

From Fig. 9, it is evident that the SMFSD-MAVOA-DNC model consistently demonstrates higher overall prediction accuracy under identical conditions. Additionally, it excels in predicting extreme values when compared to the other three models. Regarding the prediction performance for each city, it's observed that the models deliver poorer predictions for Liuan, a city with a larger standard deviation. Conversely, the prediction accuracy for Zhengzhou and Huaian, with smaller standard deviations, is relatively high. This indicates that the degree of data discretization in rainfall data has a noticeable impact on the prediction accuracy of each coupled model. However, the SMFSD-MAVOA-DNC model consistently maintains the highest performance, and the influence of data discretization on its prediction accuracy is minimal.

Table 4 demonstrates the results of rainfall prediction error comparison among the four cities, from which it can be observed that the SMFSD-MAVOA-DNC model performs the best in rainfall prediction in Zhengzhou, Linyi, Liuan and Huaian. Its average RMSE is 9.02, average MAE is 7.13, and average NSE is 0.94, which are much better than the other three models, showing higher prediction accuracy. In addition, the prediction errors of the four cities are relatively consistent, indicating that the model is more stable and representative for predicting rainfall in the Huaihe River Basin.

**Table 4:** Comparison of relative errors of SMFSD-MAVOA-DNC with other models.

Model	City	RMSE	MAE	NSE
Zhengzhou	MAVOA-DNC	18.74	15.94	0.81
	SMFSD-MAVOA-LSTM	13.07	11.06	0.86
	SMFSD-AVOA-DNC	10.16	7.88	0.92
	SMFSD-MAVOA-DNC	5.43	4.22	0.96
Linyi	MAVOA-DNC	23.14	19.77	0.80
	SMFSD-MAVOA-LSTM	17.10	13.28	0.86
	SMFSD-AVOA-DNC	17.25	12.41	0.88
	SMFSD-MAVOA-DNC	9.85	7.51	0.94
Liuan	MAVOA-DNC	26.77	19.03	0.77
	SMFSD-MAVOA-LSTM	19.53	14.43	0.84
	SMFSD-AVOA-DNC	17.67	12.56	0.88
	SMFSD-MAVOA-DNC	11.31	8.67	0.92
Huaian	MAVOA-DNC	22.46	17.59	0.80
	SMFSD-MAVOA-LSTM	17.13	12.36	0.83
	SMFSD-AVOA-DNC	15.61	11.14	0.89
	SMFSD-MAVOA-DNC	9.50	8.12	0.94
Average value	MAVOA-DNC	22.78	18.08	0.80
	SMFSD-MAVOA-LSTM	16.71	12.78	0.85
	SMFSD-AVOA-DNC	15.17	11.23	0.89
	SMFSD-MAVOA-DNC	9.02	7.13	0.94

The combined model based on MAVOA-DNC is able to predict the general trend of the monthly rainfall series, however, compared with the SMFSD-MAVOA-DNC prediction model, its overall prediction effect is poorer, which fully demonstrates that the SMFSD decomposition effectively reduces the non-stationarity of the time series with high complexity and strong nonlinearity; meanwhile, the DNC combines the advantages of the recurrent neural network and computational processing that significantly improves the memory forgetting problem of LSTM. The comparison graph shows that the SMFSD-MAVOA-DNC based on SMFSD-MAVOA-DNC predicts the effect SMFSD-MAVOA-LSTM model. Similarly, the model optimized by Tent-improved MAVOA predicts significantly better than the SMFSD-MAVOA-DNC model.

After comprehensive analysis, the combined modeling approach of SMFSD has significantly improved the learning performance of DNC. Additionally, the application of MAVOA has effectively reduced uncertainties in model construction, resulting in enhanced predictive accuracy. However, it is important to note that the model's accuracy during the flood season (May to September) is lower compared to other months, indicating that the data's dispersion has a certain impact on the predictive accuracy of all coupled models. Although the SMFSD-MAVOA-DNC model exhibits excellent predictive accuracy and is less affected by data dispersion, there is room for improvement, particularly for cities like Liuan, where rainfall data exhibits stronger dispersion.

## Conclusion

Through the comparative study of different coupled models for rainfall prediction in Huaihe River Basin, we get the following conclusions:

1. For a single nonlinear and non-stationary rainfall time series, full VMD decomposition is not suitable for practical precipitation forecasting. After conducting a comparative study, it was determined that the best prediction accuracy can be achieved by using SMFSD decomposition. When compared to traditional VMD decomposition, the SMFSD coupled model demonstrated a significant improvement, with an average RMSE reduction of 7.42, MAE reduction of 4.83, and NSE increase of 0.05 in the four cities. This unequivocally demonstrates that the SMFSD decomposition method is better suited for real-world rainfall prediction.
2. The systematic and valuable information obtained through SMFSD decomposition is applied to DNC for prediction, effectively leveraging DNC's strengths in prior learning of both long and short-term memory data. During the prediction process, multiple hyperparameters need to be configured, and the MAVOA algorithm introduced in this study provides superior optimization for machine learning models with a greater number of hyperparameters, thereby significantly reducing uncertainties in the modeling process. As a result, we achieved improved prediction results: the SMFSD-MAVOA-DNC model exhibited an average RMSE of 9.02, an average MAE of 7.13, and an average NSE of 0.94. When compared to the SMFSD-MAVOA-DNC and SMFSD-MAVOA-LSTM models, this model demonstrated the highest accuracy in rainfall prediction in the Huaihe River Basin.
3. In comparison with models proposed in other research articles, the SMFSD-MAVOA-DNC model adopts a more practical decomposition approach for rainfall prediction. It integrates multiple optimization and analytical techniques, enabling it to effectively address the challenges presented by nonlinear and non-stationary rainfall time series, while reducing uncertainties in the modeling process. This model provides a robust tool for rainfall forecasting. It is worth noting that, although the predictive performance of SMFSD-MAVOA-DNC is relatively good, there is room for improvement in predicting data with high levels of dispersion. Additionally, this paper analyzed the fitting capabilities of coupled models based on the VMD algorithm's full decomposition method. Other decomposition and denoising algorithms could be considered as subjects for future research, aiming to further enhance the accuracy and stability of rainfall prediction.

## Data Availability

Data and materials are available from the corresponding author upon request.
